# Dynamic Object Detection in Maritime Navigation Scenarios Based on Vision–Radar Fusion

**DOI:** 10.3390/s26113508

**Published:** 2026-06-02

**Authors:** Qianqian Chen, Changshi Xiao, Bowei Li

**Affiliations:** 1 School of Artificial Intelligence and Big Data, Wuhan Business University, Wuhan 430056, China; chenqq@wbu.edu.cn; 2 School of Navigation, Wuhan University of Technology, Wuhan 430060, China; 3 China Mobile Group Hubei Co., Ltd., Wuhan 420010, China; libowei@hb.chinamobile.com

**Keywords:** vision–radar fusion, dynamic object detection, intelligent navigation, multi-scale detection, deep learning

## Abstract

**Highlights:**

This study proposes a vision–radar fusion-based dynamic object detection method for maritime navigation scenarios, addressing challenges such as occlusion, scale variation, and multi-target interference. By introducing a cross-modal feature mapping mechanism and an augmented Lagrangian-based fusion strategy, the method effectively integrates complementary visual and radar information. An improved Faster R-CNN framework with optimized RPN and multi-scale training further enhances detection performance. Experimental results on the MVRD demonstrate that the proposed approach achieves high detection accuracy and strong robustness across diverse conditions, including sunny, strong glare, foggy, and densely populated environments, highlighting its potential for reliable environmental perception in intelligent maritime navigation systems.

**What are the main findings?**

- A vision–radar fusion-based dynamic object detection method is proposed, integrating cross-modal feature mapping and augmented Lagrangian optimization to enhance feature representation and consistency.
- The proposed method achieves superior detection performance on the MVRD, reaching accuracies of 88.93%, 76.86%, 74.47%, and 83.01% under sunny, strong glare, foggy, and dense traffic scenarios, respectively.

**What are the implications of the main findings?**

- The proposed fusion framework significantly improves the robustness and reliability of dynamic object detection in complex maritime environments compared with single-sensor approaches.
- The method provides an effective solution for intelligent navigation perception systems, supporting safer and more reliable autonomous maritime operations.

**Abstract:**

With the rapid development of intelligent navigation technologies, accurate dynamic object detection in complex maritime environments remains a critical challenge due to occlusion, scale variation, and multi-target interference. To address these issues, this study proposes a vision–radar fusion-based dynamic object detection method. A cross-modal feature mapping mechanism is developed to achieve deep integration of visual and radar information, and an augmented Lagrangian optimization strategy is introduced to enhance feature consistency and representation capability. Furthermore, an improved Faster R-CNN framework is designed by optimizing the region proposal network and incorporating a multi-scale training strategy to improve detection performance for objects of varying scales. Experimental results on a self-constructed MVRD show that the proposed method achieves detection accuracies of 88.93%, 76.86%, 74.47%, and 83.01% under sunny, strong illumination, foggy, and crossing-waterway conditions, respectively. These results demonstrate that the proposed approach exhibits strong robustness and stability in complex maritime environments. Overall, the method significantly improves dynamic object detection accuracy and provides effective support for reliable environmental perception in intelligent navigation systems.

## 1. Introduction

Dynamic object detection in maritime navigation scenarios is a fundamental component of environmental perception, playing a crucial role in enhancing autonomous navigation capability and ensuring navigation safety. It is also essential for applications such as maritime traffic management, collision avoidance, and search-and-rescue operations. With the rapid development of intelligent shipping and unmanned surface vehicles, building reliable perception systems has become a prerequisite for achieving autonomous navigation. In complex maritime environments, the accuracy of dynamic object detection directly affects the safety and stability of navigation decision-making and control systems. Furthermore, accurate perception also provides critical support for higher-level tasks such as multi-ship collision avoidance decision-making and autonomous navigation in uncertain environments [1,2].

In recent years, the rapid advancement of deep learning has significantly promoted the evolution of object detection methods. Early approaches mainly relied on region proposal mechanisms, such as edge-based object proposal methods [3] and selective search strategies [4], which laid the foundation for modern detection frameworks. Meanwhile, 3D object detection techniques have gradually developed by integrating geometric modeling with visual information, including methods based on 3D proposal generation [5], monocular 3D object detection [6], and geometry-based 3D bounding box estimation [7], significantly improving spatial perception capability.

With the development of deep learning, vision-based object detection methods have achieved remarkable performance in various scenarios. However, in maritime environments, these methods still face significant challenges. Factors such as water surface reflections, illumination variations, and adverse weather conditions (e.g., fog and haze) can severely degrade image quality, thereby reducing detection performance. In addition, detecting small and distant objects remains difficult, while occlusion and dynamic interference in multi-target scenarios further increase detection complexity. Therefore, relying solely on visual information is insufficient to achieve robust perception in complex maritime scenarios.

To address these challenges, multi-sensor fusion methods have attracted increasing attention, among which vision–radar fusion has emerged as a promising research direction due to its complementary advantages. Radar sensors provide reliable distance and velocity measurements and maintain robustness under adverse environmental conditions, while vision sensors offer rich semantic and texture information. Early fusion methods achieved performance improvements through joint modeling of multi-source data, such as continuous fusion strategies [8] and semantic-enhanced point cloud fusion approaches [9]. In addition, deep learning-based radar detection methods have been developed, providing an important foundation for multi-modal fusion research [10].

In recent years, vision–radar fusion has achieved significant progress in object detection. Representative work, such as CenterFusion, introduced a center-based association mechanism for cross-modal fusion [11]. Building upon this, multi-level fusion approaches have been proposed, such as RCM-Fusion, which enhances detection performance through hierarchical feature fusion [12], and MVFusion, which leverages multi-view semantic alignment for more effective information integration [13]. Meanwhile, bird’s-eye-view (BEV)-based fusion methods have become a research hotspot. For instance, RCBEVDet significantly improves detection performance by unifying spatial representations [14], its improved variant further enhances accuracy [15], and BEV-Radar introduces bidirectional fusion mechanisms to strengthen cross-modal interaction [16].

Furthermore, Transformer-based fusion models have gained increasing attention. TransCAR employs self-attention mechanisms to improve cross-modal feature interaction [17]. To address the sparsity of radar data, modality interaction and feature enhancement strategies have been proposed to improve radar representation [18]. Attention mechanisms have also been widely adopted in multi-modal fusion, where spatial attention-based methods effectively enhance feature representation capability [19]. In addition, end-to-end fusion frameworks continue to evolve, with models such as NextFusion achieving efficient fusion through attention mechanisms [20]. Meanwhile, recent studies have also explored trajectory prediction and motion modeling using advanced deep learning architectures such as Transformer, further improving spatiotemporal understanding in maritime environments [21].

In terms of model optimization, recent studies have further improved fusion performance. Deep learning-based fusion methods have been advanced in journals such as *Information Fusion* [22], while dynamic fusion and knowledge distillation strategies have been introduced to enhance robustness and generalization ability [23]. Comprehensive surveys have systematically summarized the development of vision–radar fusion and highlighted its importance in complex environments [24]. Moreover, review studies focusing on autonomous driving scenarios further demonstrate the effectiveness of multi-modal perception methods [25]. In parallel, control and planning methods for unmanned surface vehicles, including adaptive tracking control [26] and reinforcement learning-based path planning [27], have further promoted the integration of perception and decision-making.

In practical applications, multi-modal fusion methods have shown significant advantages on public datasets. For example, the nuScenes dataset provides a standard benchmark for multi-modal perception research [28], and studies have demonstrated that fusion-based methods outperform vision-only approaches under adverse weather conditions [29]. In addition, research published in Sensors has validated the effectiveness of radar–camera fusion under different representations, including perspective view and bird’s-eye view [30]. Context clustering-based fusion methods have also been proposed to improve detection performance in complex environments [31], while spatial attention fusion methods further enhance the model’s ability to focus on critical features. Furthermore, path planning methods integrating collision avoidance rules and dynamic constraints, such as those based on DWA and COLREGs, provide additional support for safe autonomous navigation [32].

Overall, vision–radar fusion technology is still in a stage of rapid development. Researchers continue to explore more effective fusion strategies, including multi-view fusion methods, multi-level fusion frameworks, and modality interaction mechanisms [33], all of which contribute to advancing the field. Deep learning-based multi-modal perception methods demonstrate strong potential for applications in complex dynamic environments.

Although recent Transformer-based [34] and BEV-based [35] fusion methods have achieved impressive performance in autonomous driving scenarios, these methods usually rely on large-scale annotated datasets, dense sensor measurements, and high computational resources. In practical maritime navigation scenarios, especially inland waterway environments, publicly available datasets remain limited, radar measurements are relatively sparse, and deployment platforms often have constrained computing resources. Moreover, the environmental characteristics of maritime scenes, such as water-surface reflections, low visibility, and dynamic wave interference, differ significantly from road traffic scenarios. Therefore, directly applying autonomous-driving-oriented fusion frameworks to maritime navigation environments remains challenging.

Despite these advances, several key challenges remain. Specifically, existing methods exhibit three primary shortcomings:

(1) insufficient cross-modal feature alignment due to the heterogeneity between radar and visual data;
(2) limited fusion capability, as some methods rely on relatively simple fusion strategies and fail to fully exploit complementary information;
(3) inadequate multi-scale detection performance, particularly for small and distant targets in complex maritime environments.

To address these issues, this paper proposes a vision–radar fusion-based dynamic object detection method for maritime navigation scenarios. By constructing an effective cross-modal feature mapping mechanism and integrating multi-scale learning strategies within an improved detection framework, the proposed method enhances detection accuracy and robustness in complex environments.

The main contributions of this work are summarized as follows:

(1) A radar–camera cooperative calibration method is proposed for vision–radar perception systems. A dedicated cooperative calibration target consisting of concentric circles and a radar corner reflector is designed to enable simultaneous acquisition of corresponding features in radar and image spaces. In addition, a recursive circle-center detection algorithm based on projective geometry and cross-ratio invariance is developed to achieve accurate calibration under perspective distortion.
(2) A vision–radar fusion framework is developed for dynamic object detection in complex maritime environments. By establishing accurate geometric correspondence between radar and camera measurements and effectively integrating heterogeneous sensor features, the proposed framework fully exploits the complementary characteristics of visual and radar information, thereby improving detection robustness and reliability.
(3) An improved Faster R-CNN detector is proposed to enhance dynamic object detection performance. By optimizing the Region Proposal Network (RPN) and introducing a feature enhancement module, the proposed detector strengthens feature interaction across different hierarchies, improves target feature representation capability, and reduces false detections and missed detections in challenging maritime scenarios.

## 2. Materials and Methods

### 2.1. Design of a Vision–Radar Fusion Calibration Model

#### 2.1.1. Radar-Camera Cooperative Target Design

**Figure 1:**
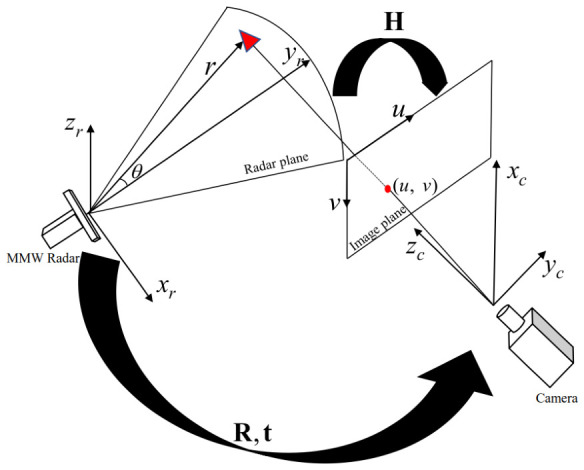
Geometry of MMW radar and camera.

As shown in Figure 1, we suppose that the radar scans in a plane, and the distance r, azimuth θ can be get from radar. Let $x_{r}={\left(x_{r},y_{r},z_{r}\right)}^{T}$ and $x_{c}={\left(x_{c},y_{c},z_{c}\right)}^{T}$ be the radar and camera coordinates, respectively, and $x={\left(u,v\right)}^{T}$ be the image plane coordinates. Using homogeneous coordinates $\tilde{x}$, ${\tilde{x}}_{r}$, ${\tilde{x}}_{c}$, the transformation can be described as follows.

(1) $$s\tilde{x}=P{\tilde{x}}_{r},P=K\left[R|t\right]$$

where ***P*** is 3 × 4 matrix which represents the transformation between the radar coordinate and the image coordinate, and ***K*** is the camera intrinsic matrix; the 3 × 3 matrix ***R*** and the 3 × 1 vector denote the rotation and translation between the sensors’ coordinates.

Since all radar data come from the radar plane, we have $z_{r}=0$, $x_{r}=rcos\theta$, and $y_{r}=rsin\theta$. Then, Equation (1) can be rewritten as follows.

(2) $$s\left[\begin{array}{c} u\\ v\\ 1 \end{array}\right]=H\left[\begin{array}{c} x_{r}\\ y_{r}\\ 1 \end{array}\right]$$

In the above equation, ***H*** is a 3 × 3 invertible homography matrix with 8 degrees of freedom, which describes the transformation between the image plane and the radar plane. The purpose of calibration is to estimate the homography matrix ***H***.

**Figure 2:**
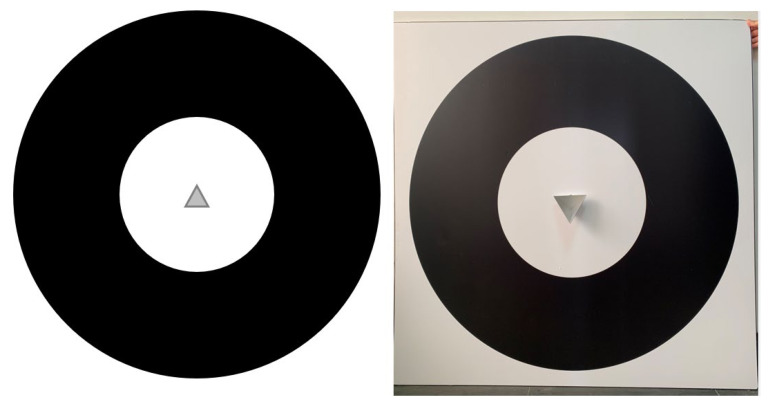
Schematic and actual diagrams of the radar camera cooperative calibration target.

To solve Equation (2), at least 4 sets of points corresponding between the radar plane and the image plane are needed. To facilitate simultaneous acquisition of target points in the radar plane and in the image, a cooperative calibration target consisting of concentric rings combined with a radar corner reflector was designed. As shown in Figure 2, the visual sign, the shape of a black ring, is formed by two concentric circles, which is convenient for the detection of the center of the circle in the image. For the purpose that the radar corner reflector is a feature for both radar and camera, the reflector is placed at the center of the circle.

#### 2.1.2. Detection of Cooperative Target

**Figure 3:**
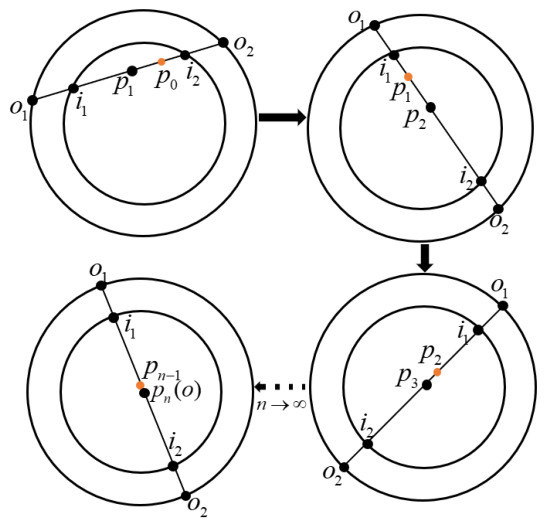
Standard concentric circle center detection process.

A recursive circle center detection algorithm is described as follows. As shown in Figure 3, a random point $p_{0}$ inside the circles is selected as the start point, and a random line is made through the start point, then the line will intersect the outer circle at the points $o_{1}$, $o_{2}$ and intersect the inner circle at the points $i_{1}$, $i_{2}$. The midpoint $p_{1}$ of $o_{1}$ and $o_{2}$ is the same as the midpoint of $i_{1}$ and $i_{2}$, which can be computed easily using Euclidean geometry. Then, take $p_{1}$ as a starting point and repeat the process above, then the new midpoint $p_{2}$ can be computed. Use $p_{2}$ as a starting point and repeat the process, so the new computed midpoint $p_{3}$ can be obtained. Execute the procedure recursively, A series of midpoints $\left\{p_{1},p_{2},\ldots ,p_{n}\right\}$ will be obtained, when n → ∞, the point $p_{n}$ will be infinitely close to the center of the circles o.

**Figure 4:**
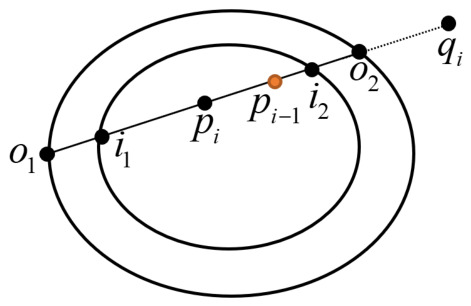
The projection of a concentric circle after imaging.

The projection of concentric circles after imaging is an ellipse, and the midpoint of the line segment is not a projection invariant, so Euclidean geometry can not be used to compute the midpoint. Just as Figure 4 shows, at the i-th recursion, we suppose that the 3D space planar points corresponding to the image points are $O_{1},I_{1},P_{i},I_{2},O_{2},Q_{2}$, where $Q_{2}$ is a vanishing point which is located at infinity, we have:

(3) $$C_{r}\left\{O_{1},O_{2};P_{i},Q_{i}\right\}=C_{r}\left\{o_{1},o_{2};p_{i},q_{i}\right\}=-1$$

(4) $$C_{r}\left\{I_{1},I_{2};P_{i},Q_{i}\right\}=C_{r}\left\{i_{1},i_{2};p_{i},q_{i}\right\}=-1\;$$

where ***Cr***{·} is the cross ratio of the four collinear points. Then, the following relation can be easily obtained:

(5) $$C_{r}\left\{o_{1},o_{2};p_{i},q_{i}\right\}=C_{r}\left\{i_{1},i_{2};p_{i},q_{i}\right\}=-1\;$$

By solving the quadratic Equation (5), the projection point of the midpoint of the line segment can be determined. For calculation convenience, random lines can be set to horizontal and vertical alternately. The center point estimation algorithm is shown in Equation (6).

After the procedure above is done, the sub-pixel coordinates of the projection point of the center of the circle are calculated. Let the obtained projection point of the center of the circle be o, then make a random line from point o, and we also have $o_{1},o_{2},i_{1},i_{2}$ corresponding to $O_{1},O_{2},I_{1},I_{2}$. According to the cross-ratio invariance, the following constraint exists.

(6) $$C_{r}\left\{o_{1},i_{1};i_{2},o_{2}\right\}=C_{r}\left\{O_{1},I_{1};I_{2}{,O}_{2}\right\}=k\;$$

where k is a known constant, which can be calculated from the geometric parameters of the cooperative target. Using (6), the noise projection of concentric circles in the image can be filtered out.

#### 2.1.3. Image Processing

After the camera acquires the image, we convert the color image into a grey-level image, and then the binary image is obtained using the Otsu algorithm. The Canny edge detector can be used to obtain the edge of the image. Then, do a morphological close operation on the image to fill the broken edges, then perform contour extraction and execute ellipse fitting on the contours using least squares method. After ellipse fitting, we suppose that the outer ellipse is $C_{1}$ with ellipse center $p_{1}$ and inner ellipse is $C_{2}$ with ellipse center $p_{2}$. To our knowledge, the center of ellipse is not the projection point of the center of the concentric circle, but we have the constraint as follows.

(7) $$\left\{\begin{array}{c} p_{1}^{T}C_{2}p_{1}<0\\ p_{2}^{T}C_{1}p_{2}<0 \end{array}\right.$$

Due to constraint (7), the noise ellipses can be filtered out, and then $p_{1}$ or $p_{2}$ can be taken as start point to figure out the projection point of the center of concentric circle. Before we install the radar corner reflector, a cross mark at the center of the calibration target is used as the ground truth.

#### 2.1.4. Homography Estimation

We suppose that the homogeneous coordinate of a point in the radar plane is $p_{r}={\;(x_{r},y_{r},1)}^{T}$, and the homogeneous coordinate of a point in the image plane is $p_{i}={\;(u,v,1)}^{T}$. Equation (2) can be rewritten as follows.

(8) $$sp_{i}=Hp_{r}$$

After the calibration target makes a linear movement in the common FOV of the camera and the radar, a series of radar points and images can be captured. Then the image sequence is processed to extract the coordinates of the center of concentric circles in each image, which are also the image coordinates of the radar reflector. Then, the least squares method was used to fit image point sequences and radar point sequences with the RANSAC method for eliminating the bad effect of accidentally extracted circle center points.

We suppose that the fitted line from the images is l and fitted line from radar plane is L. Then we have:

(9) $$l^{T}p_{i}=0,\;L^{T}p_{r}=0$$

By substituting (9) into (8), the relationship between lines in the image plane and the radar plane becomes:

(10) $$sL=H^{T}l$$

**Figure 5:**
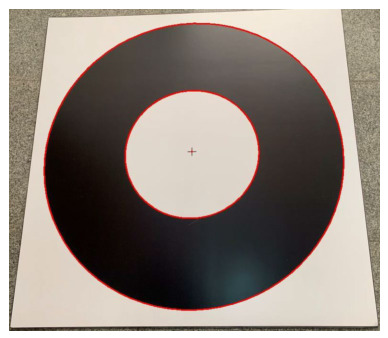
Target detection result.

At least 4 sets of line correspondences are needed to solve Equation (10). We can find that there is no need to align the timestamps of radar data and images, and thanks to the motion prediction algorithm within the radar, a linear movement of the calibration target can be easily captured by the radar. Line-based approaches perform better than point-based ones for homography estimation, but when the image lines are passing through or close to the origin, the line-based homography estimation becomes wildly unstable. The detection result is shown in Figure 5.

Given a set of lines $l_{i}={\left(a_{i},b_{i},c_{i}\right)}^{T}(i=1,2,3,\ldots ,n)$, then the transformation matrices are as follows.

(11) $$T_{1}=\left[\begin{array}{ccc} 1 & 0 & -t_{1}/t_{3}\\ 0 & 1 & -t_{2}/t_{3}\\ 0 & 0 & 1 \end{array}\right],\;T_{2}=\left[\begin{array}{ccc} 1 & 0 & 0\\ 0 & 1 & 0\\ 0 & 0 & \alpha \end{array}\right]$$

where $t_{1}=\sum_{i=1}^{n}a_{i}$, $t_{2}=\sum_{i=1}^{n}b_{i}$, $t_{3}=\sum_{i=1}^{n}c_{i}$. With $T_{1},$ we have ${l_{i}}^{\prime }=T_{1}l_{i}$ and ${l_{i}}^{\prime }={\left({a_{i}}^{\prime },{b_{i}}^{\prime },{c_{i}}^{\prime }\right)}^{T}$. Then $\alpha =\sqrt{\frac{\sum_{i=1}^{n}\left({a_{i}}^{\prime 2}+{b_{i}}^{\prime 2}\right)}{2\sum_{i=1}^{n}{c_{i}}^{\prime 2}}}$, after the transformation of ${l_{i}}^{\prime \prime }=T_{2}{l_{i}}^{\prime }$, we set ${l_{i}}^{\prime \prime }={l_{i}}^{\prime \prime }/\left\|{l_{i}}^{\prime \prime }\right\|$.

With the normalization approach above, we use ${l_{i}}^{\prime \prime }=T_{2}T_{1}l_{i}$, ${L_{i}}^{\prime \prime }={T_{2}}^{\prime }{T_{1}}^{\prime }L_{i}$ and set their Frobenius norms equal to 1. Then we have:

(12) $$s{L_{i}}^{\prime \prime }=H^{\prime T}{l_{i}}^{\prime \prime }$$

By solving (12) using the DLT method, the $H^{\prime }$ can be obtained, then using $H=T_{1}^{T}T_{2}^{T}H^{\prime }T_{1}^{\prime -T}T_{2}^{\prime -T}$, the homography between image plane and radar plane can be obtained. Like bundle adjustment always does, we design the optimization function as follows.

(13) $$H=\underset{H}{a\mathrm{rg}\min}{\left\|L-H^{T}l\right\|}^{2}$$

By using the Levenberg–Marquardt method to optimize (13), the optimized homography H can be obtained as the final calibration result.

### 2.2. Vision–Radar Feature Extraction and Fusion

#### 2.2.1. Visual Feature Extraction

**Figure 6:**
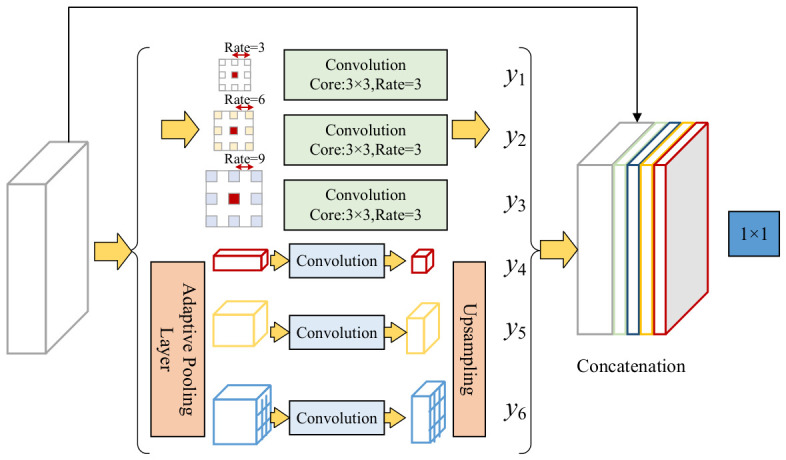
Atrous Spatial Pyramid Pooling Model.

To fully capture the global information of visual images in maritime navigation scenarios, the SPP module is employed to construct a hierarchical context pyramid pooling module for feature representation, as illustrated in Figure 6.

To enlarge the receptive field of deep networks, atrous convolutions with different dilation rates and pooling modules of varying sizes are employed and assigned to different subregions. Feature maps at different scales are upsampled and fused with atrous convolution feature maps of corresponding sizes, forming the final visual feature representation for maritime navigation scenarios. To capture global prior information and reduce information loss, a hierarchical feature network is adopted. Pyramid pooling modules at different levels upsample the feature maps to the same size as the input features and then concatenate them to construct global prior information for maritime navigation scenes.

(14) X=SP(x)×A

Here, X denotes the prior information of visual images in maritime navigation scenarios, SA(⋅) represents the spatial pyramid module, and A denotes the feature weights of the prior information of visual images in maritime navigation scenarios.

Deep feature networks are capable of extracting semantic information from maritime navigation scenes; however, when fusing visual information with radar information, it is also necessary to extract the spatial location information from visual images. To further explore the spatial positional information of visual images in maritime navigation scenarios, an attention-based fusion module is employed to enhance visual features. By learning the feature weights of both semantic and spatial information in maritime images, multiple feature maps are effectively fused, thereby improving the representation capability and robustness of visual features in maritime navigation scenarios.

The set of spatial positional information is denoted as y={(x,y)∣x=1,2,…,H; y=1,2,…,W}, where $\left(x,y\right)$ represents the spatial coordinate of a feature. The input shallow feature maps are convolved using kernels of size 1×k and k×1 to extract spatial positional information from maritime navigation scene images. The weights of the shallow spatial positional information are computed as follows:

(15) $$\left\{\begin{array}{c} C_{1}=Conv_{2}(Conv_{1}(Y,W_{1}^{1}),W_{1}^{2})\\ C_{2}=Conv_{1}(Conv_{1}(Y,W_{2}^{1}),W_{2}^{2})\\ SA=S(Y,W)=\delta (C_{1}+C_{2}) \end{array}\right.$$

Here, Conv1 and Conv2 denote convolution operations with kernel sizes of 1×k and k×1, respectively. W and the sigmoid function represents the parameters associated with spatial positional information, and SA(⋅) denotes the attention-based fusion model. Finally, the output of the shallow spatial positional information is obtained by applying weighting through SA(⋅):

(16) Y=SA(y)×B

Here, Y denotes the spatial positional features of visual images in maritime navigation scenarios, and B represents the feature weights of the spatial information of visual images in maritime navigation scenarios. The global information and spatial positional information of the visual images are processed by the spatial pyramid module (SP) and the attention module (SA), respectively, and then fused to obtain the final output of the feature fusion map *Z*.

(17) Z=SP(x)×A+SA(y)×B

#### 2.2.2. Radar Feature Extraction

Since the reflection points detected by radar for moving targets do not necessarily correspond to the geometric center of the targets, misalignment may occur during the transformation from radar detections to image pixels. As a result, radar point measurements may not accurately match the geometric contours of targets detected in visual images, making it difficult to achieve pixel-level correspondence between radar points and image objects. This issue may lead to missed detections, especially for small or non-standard targets. To address this problem, appropriate vertical and horizontal expansion of radar point maps is applied to improve the fusion accuracy between radar and visual data.

The radar observations are first projected onto a vertical plane through a projection transformation. Then, based on perspective principles, Gaussian blobs are used to expand the radar points in the image. Specifically, targets with shorter radial distances are assigned wider Gaussian distributions, while targets with higher velocities are represented with higher intensity. In this way, the radial distance and velocity information of radar-detected targets are encoded into pixel-level features and stored in the radar feature map.

(18) $$I(x,y)=I_{0}\cdot \exp(-\frac{(x-x_{0}{)}^{2}+(y-y_{0}{)}^{2}}{2\sigma^{2}})\;$$

Here, *I*(*x*, *y*) denotes the intensity at the coordinate (*x*, *y*). (*x*_0_, *y*_0_) represents the coordinates of the spot center, which are obtained by transforming the spatial coordinates of the radar-detected target into pixel coordinates through a calibration process. The parameter σ represents the width of the spot, and $I_{0}$ denotes the maximum intensity.

(19) $$\left\{\begin{array}{c} I_{0}=\frac{v}{30}\times 255\\ \sigma =\frac{d}{1500}\times 10 \end{array}\right.$$

Here, *v* represents the velocity of the radar-detected target, and *d* denotes the distance between the radar-detected target and the observation point.

**Figure 7:**
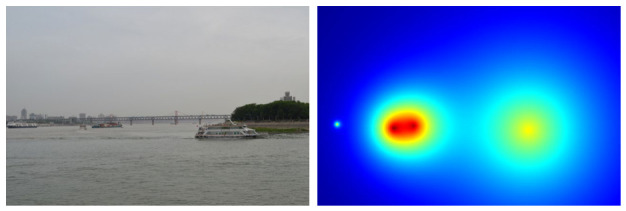
Radar Feature Map.

As shown in Figure 7, the feature maps of radar measurement points are presented. The first column shows visual images of the water navigation scene, while the second column shows the feature images of radar point traces. After expanding the velocity and distance information of radar-detected targets into feature images, they are distributed over a two-dimensional plane to generate radar feature maps.

**Figure 8:**
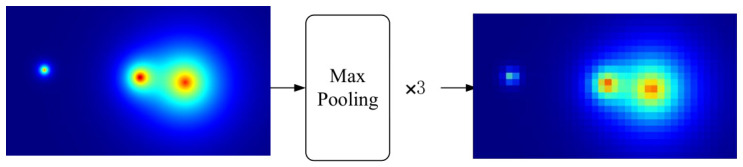
Millimeter-Wave Radar Feature Expansion Network.

The radar feature image contains two channels: the target velocity channel and the target distance channel. The size of the radar feature image is consistent with that of the visual image. Figure 8 illustrates the adopted millimeter-wave radar feature expansion network, which employs three max-pooling layers to obtain the expanded radar feature images.

Pooling operations can reduce the spatial dimensions of an image, thereby lowering computational cost. This is particularly important for models such as deep neural networks, as larger image sizes lead to increased computational and memory requirements, as well as longer training time. Pooling also helps capture salient features in images, such as edges and textures.

#### 2.2.3. Construction of a Vision–Radar Feature Image Fusion Model

Images from different sources emphasize different aspects of the same scene. For example, radar feature images focus more on capturing the dynamic information of targets, while visual images contain rich texture information. Since a single sensor cannot fully capture all the information of a scene, fusing these images can provide a more comprehensive representation of both the texture and dynamic information of water navigation scenarios. In this section, a synchronous fusion method is adopted to fuse visual and radar feature images.

There exists a mapping relationship between the fused image and the source images. Considering that visual imaging is related to light reflection, whereas radar imaging is associated with the target’s speed and heading, the fusion model treats the visual image as the data fidelity term and relies on the radar image to provide constraint information. The fidelity term can be expressed as:

(20) $$F_{1}=\frac{1}{p}{\left\|f-I\right\|}_{p}^{p}$$

Here, *f* denotes the fusion result, *I* represents the visual image, and *p* indicates the type of norm used. The constructed constraint term can be expressed as:

(21) $$F_{2}=\frac{I_{Radar}}{q}{\left\|f-R\right\|}_{q}^{q}$$

Here, $I_{Radar}$ denotes the weight of the constraint term, *R* represents the radar image, and *q* indicates the type of norm used. By adjusting the weight of the constraint term, the dynamic information from the radar image is preserved, ensuring clear target boundaries and avoiding blurring. The weighting scheme for the constraint term is modeled as:

(22) $$I_{Radar}(i,j)=\left\{\begin{array}{c} 0,x<0\\ 1-cos(p\times x),0\leq x\leq 0.5\\ 1,x>0.5 \end{array}\right.$$

Here, ${x=I}_{Radar}\left(i,j\right)-mean(I_{Radar})$, where $I_{Radar}\left(i,j\right)$ denotes the pixel value at position (*i*, *j*) in the radar image, and $mean(I_{Radar})$ represents the mean pixel value of the radar image.

To ensure that the detailed information in both the visual and radar images can be more fully integrated into the fused image—and since image details can be represented by gradients—the background region is defined as:

(23) $$F_{3}=\frac{1-I_{Radar}}{m}{\left\|D_{f}-D_{I}-D_{R}\right\|}_{m}^{m}$$

Here, D denotes the gradient operator; $D_{f},D_{I},D_{R}$ represent the gradients of the fused image, the visual image, and the radar image, respectively; and m indicates the type of norm used.

The $L_{2}$ norm is commonly used to measure image similarity, especially when the error between the fused result and the input images follows a Gaussian distribution; therefore, the $L_{2}$ norm is selected. Accordingly, for the fidelity term $F_{1}$ and the constraint term $F_{2}$, the $L_{2}$ norm is adopted (p=q=2). Since image gradients vary slowly in flat regions but change rapidly in edge or texture regions, the gradient matrix is typically sparse, containing a large number of zero values. Therefore, the background term $F_{3}\;$ adopts the more sparsity-promoting $L_{1}$ norm. Finally, the fusion task of visual and radar images is formulated as:

(24) $$F_{1}=\frac{1}{2}{\left\|f-I\right\|}_{2}^{2}+\frac{a_{1}I_{Radar}}{2}{\left\|f-R\right\|}_{2}^{2}+a_{2}(1-I_{Radar}){\left\|D_{f}-D_{I}-D_{R}\right\|}_{1}$$

Here, $a_{1}$ and $a_{2}$ are weighting parameters.

Since the L1 norm is a non-convex function that cannot be directly solved, variable splitting is adopted to address the unconstrained formulation. First, a parameter is introduced to transform the unconstrained problem into a constrained one. Then, the augmented Lagrangian function of the constrained problem is defined as follows:

(25) $$L(f,k,u)=\frac{1}{2}{\left\|f-I\right\|}_{2}^{2}+\frac{a_{1}I_{Radar}}{2}{\left\|f-R\right\|}_{2}^{2}+a_{2}(1-I_{Radar})({\left\|k\right\|}_{1}+<u,D_{f}-D_{I}-D_{R}-k>\;+\frac{r}{2}{\left\|D_{f}-D_{I}-D_{R}-k\right\|}_{2}^{2})$$

where *μ* is the Lagrange multiplier, and *ρ* > 0 is a parameter associated with the constraint. By applying variable splitting, three subproblems with respect to *f*, *μ*, and *k* can be obtained, and the iterative scheme can be expressed as follows:

(26) $$f^{i+1}=arg\underset{f}{min}L\left(f,u^{i},k^{i}\right)$$

(27) $$k^{i+1}=arg\underset{k}{min}L\left(f^{i+1},u^{i},k\right)$$

(28) $$u^{i+1}=arg\underset{u}{min}L\left(f^{i+1},u,k^{i+1}\right)$$

where i denotes the iteration index. Then, the optimal solution can be obtained by optimizing each subproblem. For the subproblem with respect to *f*, based on the first-order optimality condition, the following requirement should be satisfied:

(29) $$\left(1+w_{1}I+w_{2}(1-I)rD^{T}D)f=R+w_{1}IR+w_{2}(I-1)(D^{T}u^{i}-rD^{T}D_{I}-rD^{T}D_{R}\right)$$

The solution to the k  subproblem is a closed-form solution, which can be obtained by applying the soft-thresholding operator, i.e.,:

(30) $$k^{i+1}=arg\underset{k}{min}{\left\|k\right\|}_{1}+\frac{r}{2}{\left\|D_{f^{i+1}}-D_{I}-D_{R}+\frac{u^{i}}{r}-k\right\|}_{2}^{2}=max(D_{f^{i+1}}-D_{I}-D_{R}+\frac{u^{i}}{r},\frac{1}{r})$$

The Lagrange multiplier μ can be updated according to the following expression:

(31) $$u^{i+1}=u^{i}+r(D_{f^{i+1}}-D_{I}-D_{R}-k^{i+1})$$

### 2.3. Dynamic Object Detection Based on Fused Images

#### 2.3.1. Dynamic Object Detection Network Design

After fusing visual and radar feature images, the texture information and state information of the target are integrated, which provides significant advantages for target detection. On this basis, an improved R-CNN series algorithm is adopted to detect dynamic targets in waterborne navigation scenes. Considering the need to handle large-scale data and complex scenarios, the more efficient Faster R-CNN is employed, which not only performs object detection but also enables precise object localization.

**Figure 9:**
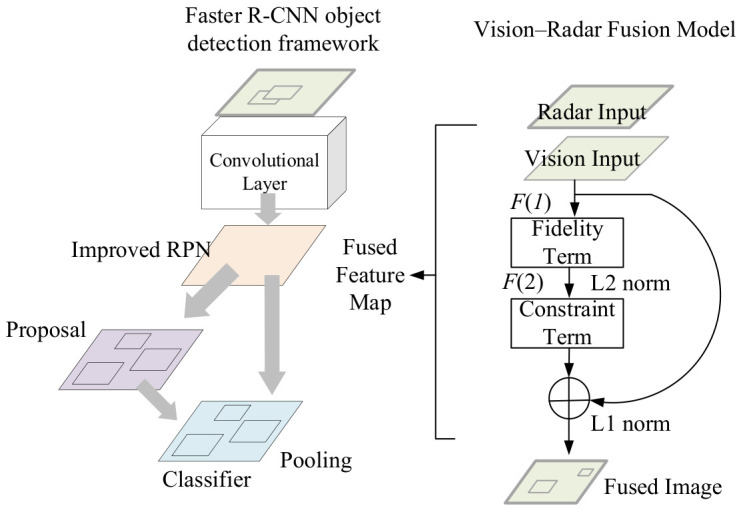
Architecture of the Improved Faster R-CNN Network.

Faster R-CNN is generally composed of three main parts: feature extraction from the input image, generation of bounding boxes, and classification with regression-based localization refinement. The overall architecture of Faster R-CNN is shown in Figure 9. The input image is first processed by a convolutional neural network to extract feature information. The resulting convolutional features are then fed into RPN to generate ROIs. The regression layer is mainly responsible for predicting the parameters of region proposals corresponding to anchor boxes, while the classification layer determines whether the object within a bounding box is a target or background.

In this section, the designed Faster R-CNN network structure incorporates an optimized RPN. As a core component of Faster R-CNN, the RPN is mainly responsible for generating candidate object regions. It operates by sliding a small network over the feature map, producing multiple anchor boxes with predefined scales and aspect ratios at each location, and predicting for each anchor whether it contains an object as well as the corresponding bounding box regression parameters.

During dynamic target detection in waterborne navigation scenes, Faster R-CNN slides anchor boxes of various scales and aspect ratios over the input feature map, thereby generating a large number of candidate regions. The RPN then selects a subset of high-quality proposals. Based on these proposals, a fully connected network performs more refined region adjustments, while a softmax classifier predicts the category of each candidate region. Finally, NMS is applied to eliminate redundant detection boxes, yielding the final detection results.

#### 2.3.2. Optimization of the RPN for Multi-Object Detection

The traditional RPN suffers from a common drawback of high computational cost. Faster R-CNN addresses this issue by applying the NMS algorithm, which retains the bounding boxes most likely to contain objects while removing other highly overlapping candidate boxes, thereby improving the efficiency of generating candidate target regions.

**Figure 10:**
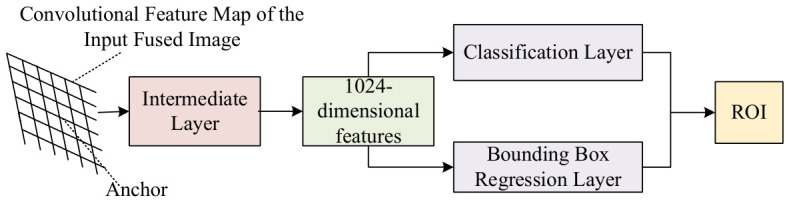
Optimized RPN Structure Diagram.

To further reduce redundancy, this paper adopts an optimized RPN model. The fused image feature map is used as input, and each anchor generates a 1024-dimensional feature vector, which is then fed into the classification and bounding box regression layers, respectively. The classification layer outputs the confidence score of the target, while the regression layer predicts the target’s location. Finally, the non-maximum suppression algorithm is applied to select high-quality candidate regions and reduce redundant detections. The overall architecture of the optimized RPN is illustrated in Figure 10.

In this paper, a 3×3 convolution kernel is employed to obtain multi-scale detection boxes. Combined with the RPN and the actual pixel size of target regions, this kernel slides over the feature map output by the feature extraction network, and the region corresponding to the center of the kernel is mapped back to the original input image.

A set of random sliding window sizes and strides is defined, and these random sliding windows are applied to images at a single scale. The sliding window is a fixed-size window that moves across the image and generates candidate boxes at each location. By adjusting the size and stride of the sliding window, detection boxes at different scales can be obtained.

#### 2.3.3. Multi-Scale Training for Multi-Object Detection Networks

The resolution of the input image has a significant impact on the detection performance of the trained model. In the feature extraction network, the generated feature maps are usually several times smaller than the original image, which makes it difficult to capture detailed features of small objects. Therefore, training with larger and more diverse images can enhance the robustness of the algorithm to a certain extent.

The use of multi-scale training can significantly improve the representational capability of the network model. Typically, a set of different scale parameters is predefined so that appropriate input image sizes can be selected as the basis during training. This approach greatly enhances the model’s robustness to objects of varying sizes.

In this study, a multi-scale training strategy is adopted for model training. Specifically, during the training process, the sample images in the training set are resized in both length and width by randomly selecting a scaling factor (a random value between 0.8 and 1.2), and then multiplying the width and height of the image by this factor. This training strategy helps improve the model’s ability to detect objects at different scales.

## 3. Experiment

### 3.1. Datasets

During the data collection phase, in order to construct a high-quality visual and radar dataset, radar and vision perception systems were deployed on a self-developed USV and a Yangtze River ferry. Data were collected from inland waterways in the Wuhan section, Donghu Lake, and open waters in Weihai, Shandong. The dataset was categorized according to scene characteristics into four types: sunny conditions, foggy conditions, strong glare, and densely populated target scenarios.

During the dataset annotation phase, the labeling mainly consists of three parts. First, the positions of targets in images are annotated using Labelbox. Second, the corresponding radar data for each target are retrieved. Third, trajectory IDs are assigned to each target in both the image and radar data.

The visual and radar dataset constructed in this study contains a total of 3848 images, 3848 frames of radar data, and 28,332 queried target objects with bounding box annotations. For the visual–radar fusion object detection experiments, the dataset is randomly divided into training, validation, and test sets. Specifically, the training set includes 2348 images and the corresponding 2348 radar frames; the validation set includes 500 images and 500 radar frames; and the test set includes 1000 images and 1000 radar frames. The dataset developed in this work is named the Marine Vision and Radar Dataset (MVRD).

### 3.2. Evaluation Indicators

In this chapter, the MVRD is used to evaluate the performance of the object detection algorithm. Precision and Recall are commonly used evaluation metrics in the field of object detection, and they are adopted here to assess the performance of the proposed algorithm. The definitions of Precision and Recall are given in Equations (32) and (33), respectively:

(32) $$Precision=\frac{TP}{TP+FP}$$

(33) $$Recall=\frac{TP}{TP+FN}$$

where *TP* denotes true positives, i.e., the number of instances correctly identified as ships; *FP* denotes false positives, i.e., the number of instances incorrectly identified as ships; and *FN* denotes false negatives, i.e., the number of missed instances.

Precision describes the proportion of correctly detected ship instances among all predicted results, and a higher Precision indicates a lower false detection rate. Recall evaluates the model’s ability to correctly identify positive instances among all actual positives, i.e., the proportion of ship instances correctly detected, and a higher Recall indicates a lower miss detection rate.

### 3.3. Ablation Experiments

#### 3.3.1. Experimental Results of Radar–Camera Object Detection

**Figure 11:**
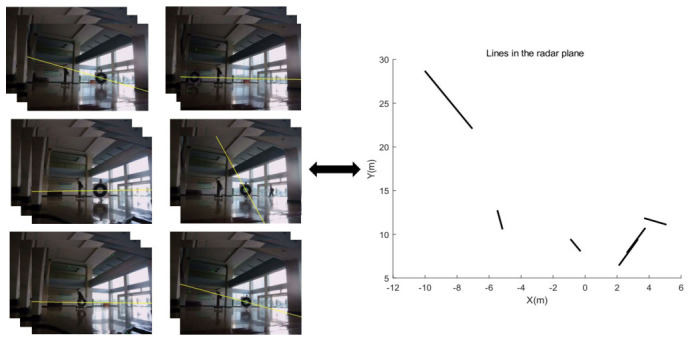
Line correspondences between the image plane and radar plane.

To establish the radar–camera correspondence, a calibration board was moved along a linear trajectory within the common field of view (FOV) of the camera and radar. During the calibration process, image sequences were captured by the camera while the radar simultaneously recorded the moving points with the highest echo intensity. After image processing, the extracted image points and radar points were independently fitted to obtain line features, from which line correspondences between the image plane and radar plane were established. As illustrated in Figure 11, the procedure was repeated more than four times to obtain sufficient correspondences for calibration.

**Figure 12:**
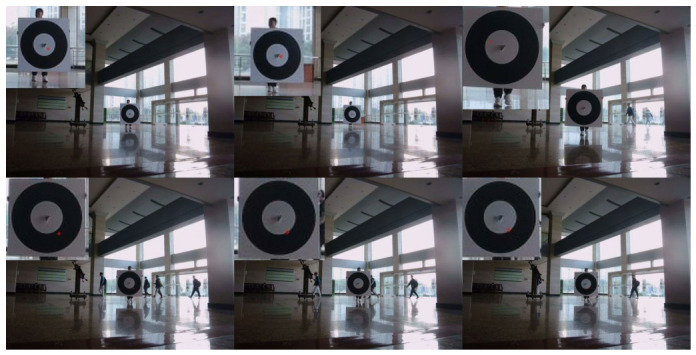
Reprojection result.

To evaluate the calibration results, a separate dataset that was not involved in the calibration process was selected for testing. Using the estimated transformation matrix, radar points were projected onto the image plane, and the reprojection results are shown in Figure 12. The projected radar points exhibit good alignment with the corresponding image features, indicating that the calibration parameters accurately describe the geometric relationship between the radar and camera.

**Figure 13:**
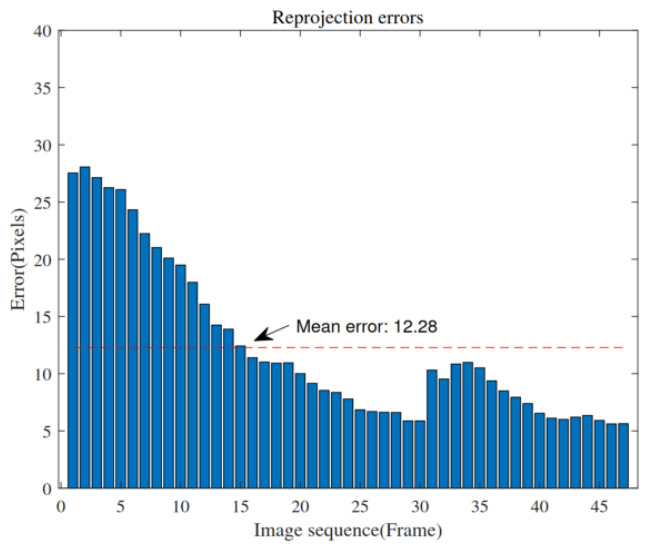
Reprojection errors in the test.

To quantitatively assess the calibration accuracy, the reprojection error was adopted as the evaluation metric:

(34) $$error=\left\|p_{i}-Hp_{r}\right\|$$

where $p_{i}$ denotes the ground-truth image point extracted from the calibration board center, $p_{r}$ represents the radar point, and H is the estimated transformation matrix. The reprojection error measures the Euclidean distance between the projected radar point and the corresponding image point. Smaller reprojection errors indicate higher calibration accuracy. The distribution of reprojection errors is presented in Figure 13, demonstrating that the proposed calibration method achieves satisfactory accuracy.

**Figure 14:**
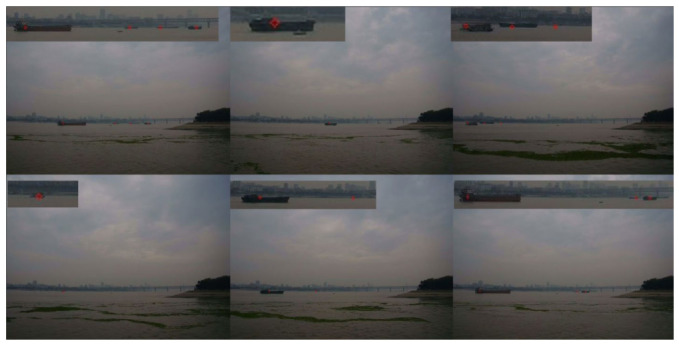
Detection results of distant moving ships.

**Figure 15:**
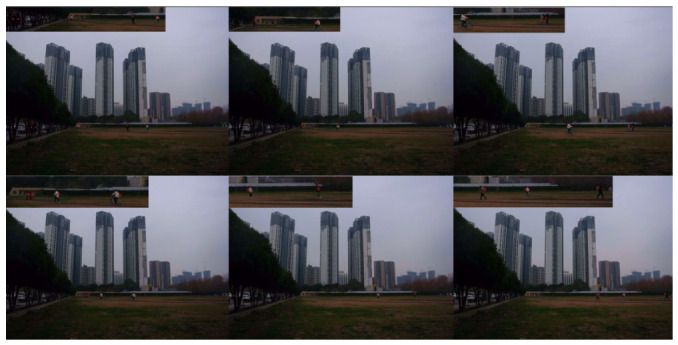
Pedestrian detection results.

To further validate the effectiveness of the calibration in practical applications, radar-assisted object detection experiments were conducted after calibration. In a maritime scene, moving ships located approximately 1.5 km from the sensor platform were successfully detected and tracked, as shown in Figure 14. In addition, pedestrian targets were accurately detected in terrestrial scenarios, as illustrated in Figure 15.

#### 3.3.2. Comparison of Ablation Experiment Results for Vision and Radar

**Figure 16:**
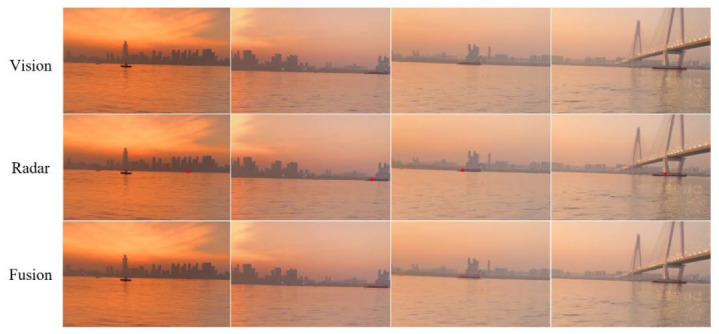
Test Results of Different Input Data in Strong Glare Scenarios.

**Figure 17:**
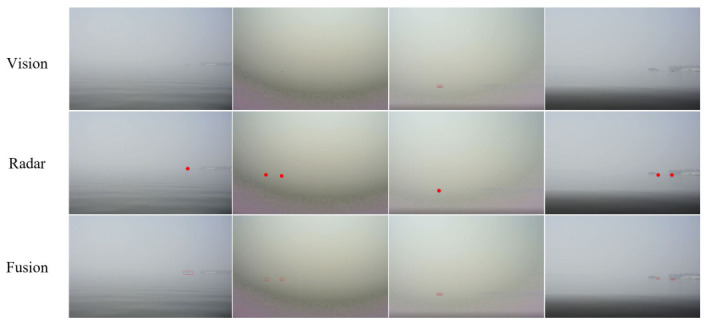
Test Results of Different Input Data in Foggy Scenarios.

**Figure 18:**
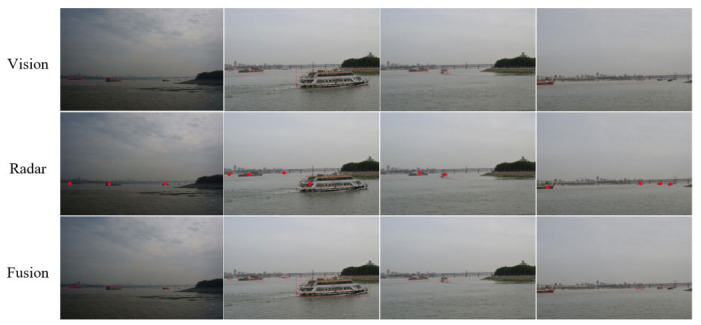
Test Results of Different Input Data in Densely Populated Target Areas.

To verify the effectiveness of the proposed vision–radar fusion model, ablation experiments were conducted on the validation subset of the MVRD using object detection algorithms based on single-sensor data sources (vision-only and radar-only). The experimental results for the three challenging scenarios are shown in Figure 16, Figure 17 and Figure 18, respectively.

The quantitative experimental results of different input data in three scenarios strong glare, foggy conditions, and densely populated target areas are shown in Table 1.

The results show that in the strong glare scenario, Precision and Recall are improved by 27.47% and 24.22%, respectively, compared with the visual sensor, and by 4.67% and 9.65%, respectively, compared with the radar sensor. In the foggy scenario, Precision and Recall are improved by 32.73% and 31.35%, respectively, compared with the visual sensor, and by 1.2% and 8.55%, respectively, compared with the radar sensor. In densely populated target scenarios, Precision and Recall are improved by 27.1% and 22.97%, respectively, compared with the visual sensor, and by 4.65% and 6.5%, respectively, compared with the radar sensor.

In strong glare scenarios, visual sensors are prone to overexposure, which may render targets invisible or difficult to recognize. Radar sensors are not affected by lighting conditions; however, they may mistakenly identify flying objects such as waterfowl as moving vessels. The fusion-based method utilizes radar data to compensate for illumination issues in visual sensing, while visual data help reduce false alarms from radar, thereby improving the reliability and accuracy of target detection under strong glare conditions.

In foggy scenarios, visual sensors may be affected by haze or raindrops, resulting in degraded image quality and increased difficulty in target detection. Radar sensors, on the other hand, are not affected by fog and can provide reliable detection data. The fusion-based method combines radar and visual data to overcome the limitations of visual sensing in foggy conditions, thereby enhancing detection robustness.

In densely populated target areas, multiple objects may overlap or be closely spaced, increasing the difficulty of detection. Radar sensors provide additional distance and velocity information, which helps distinguish and track multiple targets. The fusion-based approach can effectively handle multi-target detection and provide more accurate target location and motion information.

Therefore, compared with single-sensor-based methods, the vision–radar fusion-based dynamic object detection approach for waterborne navigation scenarios demonstrates significant advantages under challenging conditions such as strong glare, fog, and densely populated target environments, as it leverages complementary information from both sensors to improve detection performance and robustness.

#### 3.3.3. Comparison of Ablation Study Results for the Improved Faster R-CNN

To verify the effectiveness of the improved Faster R-CNN model proposed in this chapter, ablation experiments were conducted on the MVRD validation set by comparing the conventional Faster R-CNN with the improved Faster R-CNN. The results are shown in Table 2.

The results demonstrate that the improved Faster R-CNN significantly enhances the performance of dynamic object detection. In the strong glare scenario, Precision and Recall are improved by 2.85% and 7.96%, respectively. In the foggy scenario, Precision and Recall are increased by 5.22% and 3.85%, respectively. In densely populated target scenarios such as intersections, Precision and Recall are improved by 1.2% and 1.92%, respectively.

These results verify that the proposed improved Faster R-CNN enhances dynamic object detection performance through RPN optimization. Table 2 presents the comparison results of the ablation study for the improved Faster R-CNN.

### 3.4. Comparative Experiments

**Figure 19:**
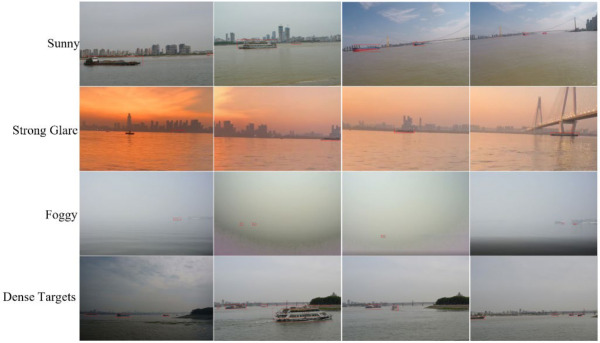
Test Results of the Algorithm on the MVRD Test Set.

Using the proposed algorithm, a quantitative evaluation of ship detection was conducted on the test subset of the MVRD. As shown in Figure 19, the first row presents the detection results in sunny conditions, the second row shows the results under strong glare, the third row corresponds to foggy conditions, and the fourth row illustrates the results in densely populated target scenarios.

By analyzing the dynamic target detection results across different scenarios, it can be observed that in sunny conditions, both visual and radar sensors can provide high-quality data. Visual sensors are capable of capturing high-resolution images under sufficient illumination, while radar can detect the position and velocity of targets regardless of lighting conditions. Therefore, the vision–radar fusion-based object detection algorithm performs well in sunny scenarios and can accurately detect moving targets.

In strong glare scenarios, visual sensors may suffer from overexposure or light spot interference, whereas radar is generally unaffected. The fusion-based algorithm can leverage radar data to compensate for illumination issues in visual data, thereby improving detection performance. By integrating information from both sensors, the algorithm achieves more accurate target detection while reducing the impact of lighting disturbances.

In foggy conditions, reduced visibility limits the performance of visual sensors, while radar can still effectively detect targets. The vision–radar fusion algorithm utilizes radar data to compensate for the limitations of visual sensing, thus enhancing the robustness of target detection. By combining information from both sensors, the algorithm mitigates the adverse effects of fog on dynamic target detection.

In densely populated target scenarios, multiple objects may overlap or be located close to each other, increasing the complexity of detection. The vision–radar fusion algorithm can utilize radar data to provide additional distance and velocity information, which helps distinguish and track multiple targets more effectively.

To quantitatively evaluate the performance of the object detection algorithm, Precision was adopted as the evaluation metric on the MVRD test set. The detection results of different dynamic object detection algorithms were assessed across four scenarios. Table 3 presents the comparison results between the proposed dynamic object detection algorithm and other object detection methods.

Table 3 compares the results of the algorithm proposed in this section with those of other ship detection algorithms. Overall, the proposed method outperforms most existing algorithms in terms of both Precision and Recall.

In the sunny scenario, the proposed algorithm exceeds Mono3D [6] by 2.14% in Precision. In the strong glare scenario, it surpasses 3D-Deepbox [7] by 3.74%. In the foggy scenario, the Precision is 3.22% higher than that of 3D-Deepbox [7]. In dense traffic scenarios such as intersections, the proposed method achieves a 3.81% improvement in Precision over 3D-Deepbox [7].

In summary, the vision–radar fusion-based dynamic object detection algorithm for waterborne navigation scenarios demonstrates strong robustness across various weather conditions and environments. By effectively integrating data from both sensors, the proposed method improves detection accuracy and reliability while reducing the impact of environmental conditions on algorithm performance.

## 4. Discussion

The experimental results demonstrate that the proposed framework achieves reliable and robust performance for dynamic object detection in complex maritime environments. The effectiveness of the proposed method can be analyzed from three aspects: radar–camera cooperative calibration, vision–radar feature fusion, and the improved detection network.

First, the proposed radar–camera cooperative calibration method provides a reliable foundation for subsequent cross-modal information fusion. Unlike conventional calibration approaches that rely on manually selected correspondences or independent feature extraction in different sensor domains, a dedicated cooperative calibration target consisting of concentric circles and a radar corner reflector is designed. This configuration enables simultaneous acquisition of corresponding features in both radar and image spaces, thereby reducing correspondence ambiguity and improving calibration consistency. Furthermore, a recursive circle-center detection algorithm based on projective geometry is developed to accurately estimate the center of concentric circles under perspective distortion. By exploiting the invariance of the cross-ratio, the proposed method achieves accurate center localization and suppresses noise interference during feature extraction. The low reprojection errors obtained in the experiments verify the accuracy of the calibration model. Moreover, the successful detection and tracking of moving ships at distances of approximately 1.5 km further demonstrates the practical applicability of the proposed calibration framework in real-world radar–camera collaborative perception tasks.

Second, the ablation experiments clearly validate the effectiveness of the proposed vision–radar fusion strategy. Compared with vision-only and radar-only approaches, the fusion framework consistently achieves superior detection performance across all evaluated scenarios. This improvement can be attributed to the complementary characteristics of the two sensing modalities. Visual images provide rich texture and semantic information, while radar measurements offer reliable distance and motion information that are less affected by illumination variations and adverse weather conditions. Through accurate sensor alignment and effective cross-modal feature integration, the proposed fusion strategy enhances perception completeness and detection reliability. The consistent improvements observed under sunny, foggy, strong-glare, and dense-traffic conditions indicate that the proposed fusion framework possesses strong robustness and environmental adaptability.

Third, the improved Faster R-CNN detector further enhances target detection performance in complex maritime environments. By optimizing the Region Proposal Network (RPN) and introducing a feature enhancement module, the detector strengthens information interaction among different feature hierarchies and improves feature representation capability. The improvements in Precision, Recall, and mAP demonstrate that the proposed network effectively reduces false detections and missed detections. In particular, the superior performance under degraded visual conditions indicates that the proposed feature enhancement strategy can preserve discriminative target information and improve the robustness of target perception. These results confirm that the improved detector plays an important role in enhancing the overall performance of the proposed framework.

Despite these promising results, several limitations remain. First, the proposed framework still relies on accurate calibration between radar and camera systems, and calibration errors may affect subsequent fusion performance. Second, although the improved Faster R-CNN achieves satisfactory detection accuracy, its computational complexity remains relatively high compared with lightweight one-stage detectors. Third, the current dataset mainly covers typical maritime scenarios and does not fully represent extreme environmental conditions.

Future work will focus on developing more advanced cross-modal fusion mechanisms, such as attention-based and Transformer-based architectures, to further improve feature interaction between heterogeneous sensors. In addition, lightweight network architectures and model compression techniques will be explored to improve real-time performance. Expanding the dataset to include more diverse maritime environments and extreme weather conditions will also help further evaluate and improve the robustness and generalization capability of the proposed framework.

## Figures and Tables

**Table 1:** Comparison of Ablation Experiment Results for Vision–Radar Fusion.

Input Data	Strong Glare		Foggy		Dense Targets	
	Precision	Recall	Precision	Recall	Precision	Recall
Visual Data	47.39%	54.88%	41.74%	44.56%	55.91%	59.83%
Radar Data	72.19%	69.45%	73.27%	67.36%	78.36%	76.93%
Fusion Data	76.86%	79.10%	74.47%	75.91%	83.01%	82.80%

**Table 2:** Comparison of Ablation Study Results for the Improved Faster R-CNN.

Detection Method	Strong Glare		Foggy		Dense Targets	
	Precision	Recall	Precision	Recall	Precision	Recall
Conventional Faster R-CNN	74.01%	71.14%	69.25%	72.06%	81.81%	80.88%
Improved Faster R-CNN	76.86%	79.1%	74.47%	75.91%	83.01%	82.80%

**Table 3:** Comparison of Precision for Dynamic Object Detection Results.

Detection Method	Sunny	Strong Glare	Foggy	Dense Targets
SS [3]	71.58%	62.41%	58%	63.97%
EB [4]	74.86%	67.23%	66.80%	66.79%
3DOP [5]	82.47%	68.75%	63.68%	71.56%
Mono3D [6]	86.79%	71.56%	70.03%	72.07%
3D-Deepbox [7]	84.12%	73.12%	71.25%	79.28%
Proposed Method	**88.93%**	**76.86%**	**74.47%**	**83.01%**

## Data Availability

The data presented in this study are available from the corresponding author upon reasonable request.

## Abbreviations

The following abbreviations are used in this manuscript:

R-CNN	Region-based Convolutional Neural Networks
DWA	Dynamic Window Approach
COLREGs	International Regulations for Preventing Collisions at Sea
MMW Radar	Millimeter Wave Radar
USV	unmanned surface vehicle
SPP	Spatial Pyramid Pooling
ASPP	Atrous Spatial Pyramid Pooling
FOV	Field of View
RANSAC	Random Sample Consensus
DLT	Direct Linear Transformation
Faster R-CNN	Faster Region-based Convolutional Neural Network
RPN	Region Proposal Network
NMS	Non-maximum Suppression
ROI	Region of Interest
MVRD	Marine Vision and Radar Dataset
TP	True Positive
FP	False Positive
FN	False Negative

## References

[1] Zhang K., Huang L., He Y., Wang B., Chen J., Tian Y., Zhao X. (2023). A real-time multi-ship collision avoidance decision-making system for autonomous ships considering ship motion uncertainty. Ocean Eng..

[2] Zhao X., Huang L., Zhang K., Mou J., Yu D., He Y. (2024). Dynamic adaptive decision-making method for autonomous navigation of ships in coastal waters. IEEE Trans. Intell. Transp. Syst..

[3] Zitnick C., Dollár P. Edge boxes. Locating object proposals from edges. Proceedings of the Computer Vision–ECCV 2014: 13th European Conference.

[4] van de Sande K.E.A., Uijlings J.R.R., Gevers T., Smeulders A.W.M. Segmentation as Selective Search for Object Recognition. Proceedings of the IEEE International Conference on Computer Vision.

[5] Chen X., Kundu K., Zhu Y., Ma H., Fidler S., Urtasun R. (2016). 3D Object Proposals for Accurate Object lass Detection. arXiv.

[6] Chen X., Kundu K., Zhang Z., Ma H., Fidler S., Urtasun R. Monocular 3d object detection for autonomous driving. Proceedings of the IEEE Conference on Computer Vision and Pattern Recognition.

[7] Mousavian A., Anguelov D., Flynn J., Kosecka J. 3d bounding box estimation using deep learning and geometry. Proceedings of the IEEE Conference on Computer Vision and Pattern Recognition.

[8] Liang M., Yang B., Wang S., Urtasun R. Deep continuous fusion for multi-sensor 3d object detection. Proceedings of the European Conference on Computer Vision (ECCV).

[9] Vora S., Lang A.H., Helou B., Beijbom O. Pointpainting: Sequential fusion for 3d object detection. Proceedings of the IEEE/CVF Conference on Computer Vision and Pattern Recognition.

[10] Major B., Fontijne D., Ansari A., Sukhavasi R.T., Gowaikar R., Hamilton M. Vehicle detection with automotive radar using deep learning on range-azimuth-doppler tensors. Proceedings of the IEEE/CVF International Conference on Computer Vision Workshops.

[11] Nabati R., Qi H. Centerfusion: Center-based radar and camera fusion for 3d object detection. Proceedings of the IEEE/CVF Winter Conference on Applications of Computer Vision.

[12] Kim J., Seong M., Bang G., Kum D., Choi J.W. Rcm-fusion: Radar-camera multi-level fusion for 3d object detection. Proceedings of the 2024 IEEE International Conference on Robotics and Automation (ICRA).

[13] Wu Z., Chen G., Gan Y., Wang L., Pu J. (2023). Mvfusion: Multi-view 3d object detection with semantic-aligned radar and camera fusion. arXiv.

[14] Lin Z., Liu Z., Xia Z., Wang X., Wang Y., Qi S., Dong Y., Dong N., Zhang L., Zhu C. RCBEVDet: Radar-camera fusion in bird’s eye view for 3D object detection. Proceedings of the IEEE/CVF Conference on Computer Vision and Pattern Recognition.

[15] Lin Z., Liu Z., Wang Y., Zhang L., Zhu C. (2024). RCBEVDet++: Toward high-accuracy radar-camera fusion 3D perception network. arXiv.

[16] Zhao Y., Zhang L., Deng J., Zhang Y. (2024). BEV-radar: Bidirectional radar-camera fusion for 3D object detection. JUSTC.

[17] Pang S., Morris D., Radha H. TransCAR: Transformer-based camera-and-radar fusion for 3D object detection. Proceedings of the 2023 IEEE/RSJ International Conference on Intelligent Robots and Systems.

[18] Liu X., Li Z., Zhou Y., Peng Y., Luo J. (2024). Camera–radar fusion with modality interaction and radar gaussian expansion for 3D object detection. Cyborg Bionic Syst..

[19] Chang S., Zhang Y., Zhang F., Zhao X., Huang S., Feng Z., Wei Z. (2020). Spatial attention fusion for obstacle detection using mmwave radar and vision sensor. Sensors.

[20] Kalgaonkar P., El-Sharkawy M. (2024). Nextfusion: Attention-based camera-radar fusion network for improved three-dimensional object detection and tracking. Future Internet.

[21] Chen X., Wu P., Wu Y., Aboud L., Postolache O., Wang Z. (2025). Ship trajectory prediction via a transformer-based model by considering spatial-temporal dependency. Intell. Robot..

[22] Wu Y. (2025). Fusion-based modeling of an intelligent algorithm for enhanced object detection using a deep learning approach on radar and camera data. Inf. Fusion.

[23] Wang J., Du C., Ge T., Liu B., Xiong S. (2025). D3PD: Dual distillation and dynamic fusion for camera-radar 3D perception. Pattern Recognit..

[24] Shi K., He S., Shi Z., Chen A., Xiong Z., Chen J., Luo J. (2025). Radar and camera fusion for object detection and tracking: A comprehensive survey. IEEE Commun. Surv. Tutor..

[25] Yao S., Guan R., Huang X., Li Z., Sha X., Yue Y., Lim E.G., Seo H., Man K.L., Zhu X. (2023). Radar-camera fusion for object detection and semantic segmentation in autonomous driving: A comprehensive review. IEEE Trans. Intell. Veh..

[26] Zhang J., Liu X., Wang X., Wang Y., Wang Y. (2024). Adaptive prescribed performance tracking control for underactuated unmanned surface ships with input quantization. Intell. Robot..

[27] Hua M., Zhou W., Cheng H., Chen Z. (2024). Improved DDPG algorithm-based path planning for unmanned surface vehicles. Intell. Robot..

[28] Caesar H., Bankiti V., Lang A.H., Vora S., Liong V.E., Xu Q., Krishnan A., Pan Y., Baldan G., Beijbom O. nuscenes: A multimodal dataset for autonomous driving. Proceedings of the IEEE/CVF Conference on Computer Vision and Pattern Recognition.

[29] Bijelic M., Gruber T., Mannan F., Kraus F., Ritter W., Dietmayer K., Heide F. Seeing through fog without seeing fog: Deep multimodal sensor fusion in unseen adverse weather. Proceedings of the IEEE/CVF Conference on Computer Vision and Pattern Recognition.

[30] Xiao Y., Chen X., Wang Y., Fu Z. (2025). Radar–Camera Fusion in Perspective View and Bird’s Eye View for 3D Object Detection. Sensors.

[31] He W., Deng Z., Ye Y., Pan P. (2023). ConCs-Fusion: A Context Clustering-Based Radar and Camera Fusion for Three-Dimensional Object Detection. Remote Sens..

[32] Liu S., Wang X., Wu Y., Li Q., Yan J., Levin E. (2024). Path planning method for USVs based on improved DWA and COLREGs. Intell. Robot..

[33] Dosovitskiy A., Beyer L., Kolesnikov A., Weissenborn D., Zhai X., Unterthiner T., Dehghani M., Minderer M., Heigold G., Gelly S. (2020). An image is worth 16x16 words: Transformers for image recognition at scale. arXiv.

[34] Chu F., Li H., Xie L., Zhao J. (2025). A survey of transformer architectures for autonomous driving. Expert Syst. Appl..

[35] Li H., Zhao Y., Zhong J., Wang B., Sun C., Sun F. (2025). Delving into the secrets of BEV 3D object detection in autonomous driving: A comprehensive survey. IEEE Trans. Intell. Transp. Syst..

